# The photopic negative response (PhNR): measurement approaches and utility in glaucoma

**DOI:** 10.1007/s10792-020-01515-0

**Published:** 2020-07-31

**Authors:** Matteo Prencipe, Tommaso Perossini, Giampaolo Brancoli, Mario Perossini

**Affiliations:** 1 Department of Surgical, Medical, Molecular Pathology and of Critical Area, University of Pisa, Pisa, Italy; 2 Studio Oculistico Associato Mario and Tommaso Perossini, Livorno, Italy

**Keywords:** PhNR, Photopic negative response, Retinal ganglion cell, Glaucoma, ERG, Electroretinogram

## Abstract

**Purpose:**

Visual electrophysiological testing continues to generate interest among glaucoma experts because of its potential help in clarifying disease pathophysiology and promoting early detection of glaucomatous damage. The photopic negative response (PhNR) is a slow negative component of the full-field electroretinogram that has been shown to provide specific information about retinal ganglion cells (RGCs) activity. The purpose of this article is to review the literature to explore the currently available measurement methods and the utility of PhNR in glaucoma diagnostic process.

**Methods:**

We gathered publications related to the origins, types of stimuli used, measurements methods and applications of the PhNR of ERG in animal models and humans through a search of the literature cited in PubMed. Search terms were: *“PhNR”, “photopic negative response”, “glaucoma”, “glaucomatous optic neuropathy”, “ERG”, “electroretinogram”.*

**Results:**

The most reliable PhNR measurements are obtained using a red stimulus on a blue background, without requiring refractive correction, fixation monitoring, or ocular media transparency. Given its direct correlation with RGCs response, the PhNR measured as baseline-to-trough (BT) represents the most reliable parameter of evaluation. Glaucoma patients with evident perimetric defects show pathologic PhNR values. Even though the PhNR is promising in detecting early RGCs impairment, distinguishing between healthy subjects and suspect patients at risk of developing glaucomatous damage still remains challenging.

**Conclusion:**

The PhNR is a useful additional tool to explore disorders that affect the innermost retina, including glaucoma and other forms of optic neuropathy. In particular, comparing reports of the standard examinations (optic disc assessment, OCT RNFL measurement, standard automated perimetry) with the results of electrophysiological tests may be helpful in solving clinical diagnostic and management dilemmas. On the one hand, the PhNR of the ERG can examine the parvocellular pathways; on the other hand, the steady-state pattern ERG optimized for glaucoma screening (PERGLA) can explore the magnocellular pathways. This could give ophthalmologists a useful feedback to identify early RGCs alterations suggestive of glaucoma, stratify the risk and potentially monitor disease progression.

## Introduction

Electroretinography is a minimally invasive diagnostic test that detects the electrical response of the retina to photic stimulation, which usually consists of a brief flash of light. Most often, electroretinograms (ERGs) are recorded using electrodes at the surface of the eye, which measures a summation of electrical activity of different retinal cells at corneal level [1].

**Fig. 1:**
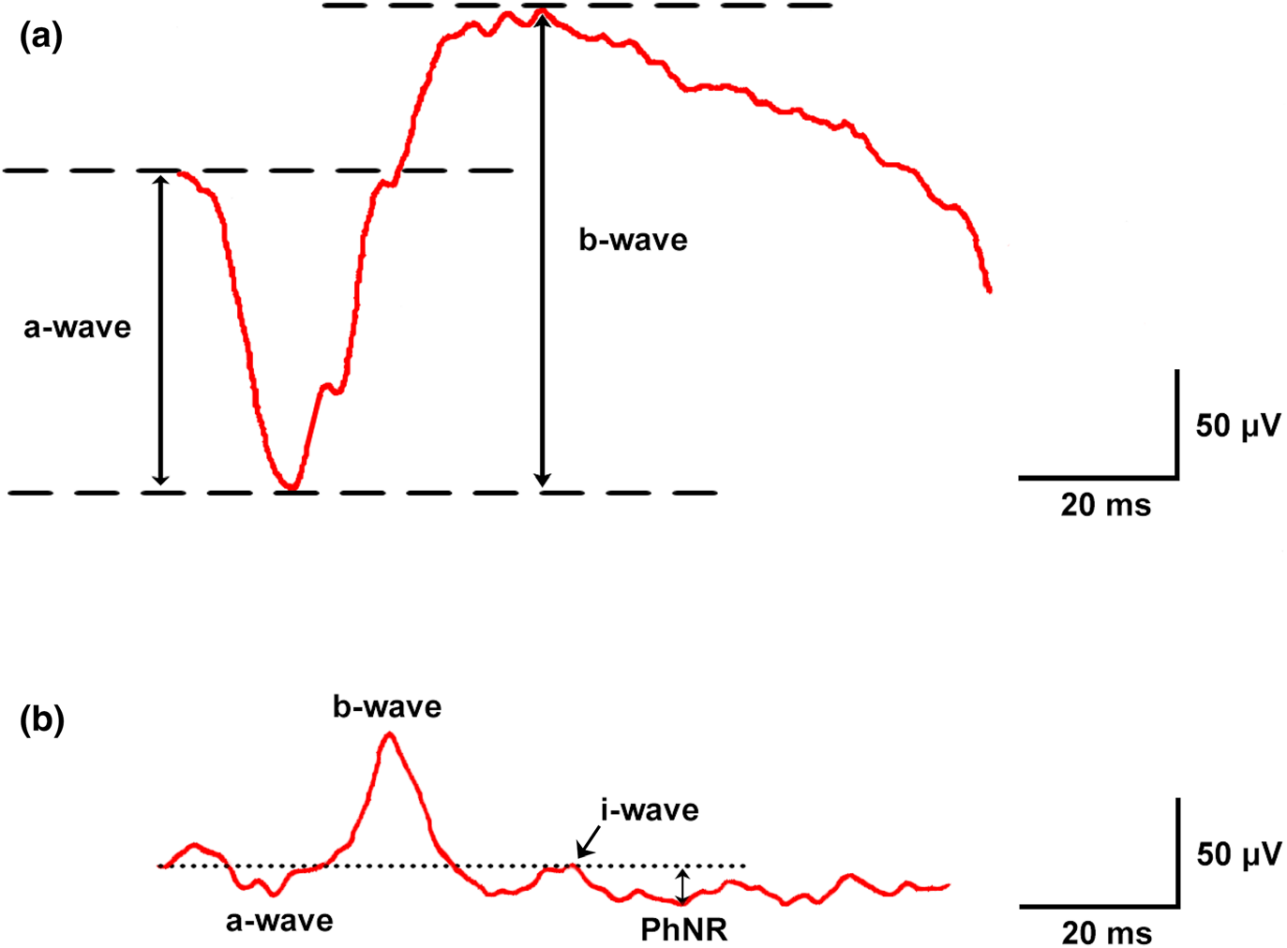
Illustration of the dark- and light-adapted full-field electroretinogram (ERG) in a male subject. A standard flashlight elicits a mass electrical response of the entire retina. **a** Under scotopic conditions (white stimulus 10 cd s/m^2^), the a-wave is a negative deflection generated mostly by the rods, while the following positive b-wave derives predominantly from Müller and ON-bipolar cells. **b** In photopic conditions (white flash 3 cd s/m^2^ on white background 30 cd/m^2^), rods are saturated and the a-wave arises from cone photoreceptors and cone OFF-bipolar cells, whereas the b-wave results from ON- and OFF-cone bipolar cells. Although less evident with standard W/W stimulation, a positive i-wave and a negative PhNR may also be noted

The flashlight stimulates retinal photoreceptors at the beginning of the visual pathway, eliciting a biphasic waveform whose main components are represented by the a- and b-waves. Under scotopic conditions, the a-wave is a negative deflection generated mostly by the rods [2], while the following positive b-wave results from the electrical activity of depolarizing bipolar cells and the dependent bipolar [K+] currents that affect the Müller cells [3, 4] (Fig. 1a). In photopic conditions, when rod responses are saturated, the ERG reflects the complex interaction and activity of the cone circuits cells: the a-wave is generated by the cone photocurrents [5–7] with additional contributions from the hyperpolarizing cone bipolar cells and the horizontal cells [8]; on the other hand, the b-wave results from the combined activity of depolarization and hyperpolarization of the bipolar cells (“ON” and “OFF”, respectively), horizontal cells and Müller cells [9] (Fig. 1b).

**Fig. 2:**
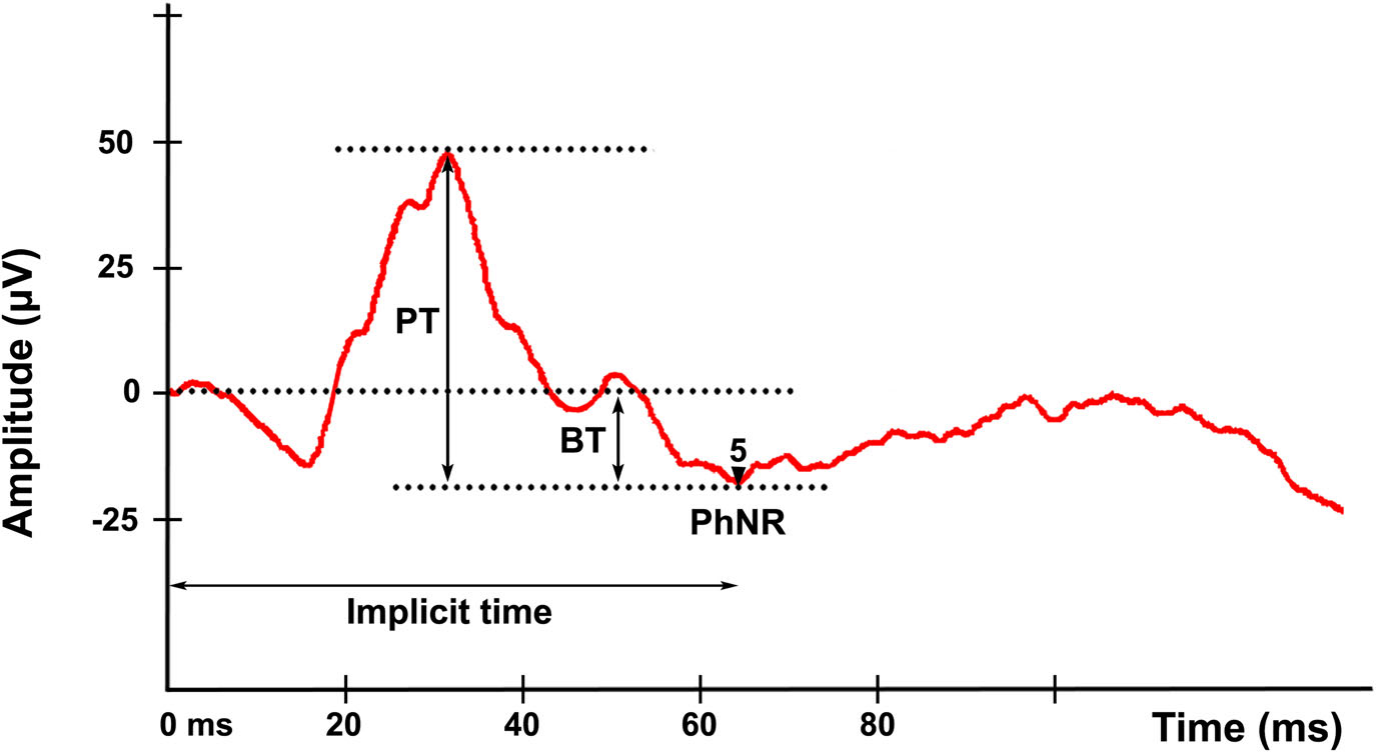
Illustration of the photopic negative response (PhNR) of the ERG recorded following ISCEV guidelines (red light stimulus < 5 ms, 2.5 cd s/m^2^ on blue background 10 cd/m^2^) in a male subject. The photopic negative response (PhNR) is a slow negative component that follows the b-wave and represents the electrical activity of the retinal ganglion cell (RGC) layer. Figure shows PhNR amplitude measurements from baseline to PhNR trough (BT) and from b-wave peak to PhNR trough (PT). Implicit time refers to the interval between the stimulation and the peak of the negative wave

Conceptually, it was believed that the ERG trace merely represented the manifestation of the electric potentials of photoreceptors and bipolar cells, conveying these signals to the Müller cells, a supporting scaffold with extensions that cross the entire retina. However, it was noted later that the retinal ganglion cells (RGCs) contributed to the photopic (cone-driven) ERG in the form of a photopic negative response (PhNR) [10–12] (Fig. 1b). The PhNR is a slow negative component that follows the b-wave of the photopic ERG and represents the functional status of the inner layers of the retina (Fig. 2). More specifically, it originates in the RGC layer from the electrical activity of RGCs themselves and, given its slow timing, includes amacrine and glial cells mediation and contribution. Confirming this, intravitreal injection of tetrodotoxin in non-human primates inhibits the action potentials of RGCs and amacrine cells, subsequently reducing PhNR amplitude and prolonging implicit time [11, 13–15]. Therefore, considering that glaucoma occurs as a result of the degeneration of the RGCs and their axons, the PhNR may be helpful for the detection of early glaucomatous damage.

It is worth mentioning that, using long-lasting stimuli that dissociate the ON from the OFF response, a PhNR appears both after the b-wave (PhNR relative to the ON-pathways) and after the d-wave (PhNR relative to the OFF-pathways). Moreover, when employing this type of stimulation, a positive i-wave after both b- and d-waves is observed. The i-wave recorded in the conventional short-flash ERG, which we usually observe before the PhNR, represents the combined activity of the ON- and OFF-bipolar cell pathways [16]. Nevertheless, the exact origin of i-waves has not been completely clarified. Rosolen et al. claimed a genesis from the innermost retinal layers [17], while other Authors indicated a more distal origin in the OFF-pathways [11, 14]*.* Interestingly, Horn et al. reported increased amplitudes of OFF i-waves in glaucomatous subjects [16], which was in agreement with previous findings by Viswanathan et al. in primary open-angle glaucoma patients [18], and by Rangaswamy et al. in glaucoma animal models [14]. Horn et al. also suggested that a reduction in PhNR amplitude could bring out the i-wave in glaucoma [16].

The purpose of this article is to review the different types of stimuli and measurements methods that may be used to assess the PhNR in a clinical setting, focusing on glaucomatous disease evaluation.

## Measuring the PhNR: types of light stimulation

Among the different ERG waves, PhNR was first reported by Viswanathan et al., who used brief (≤ 5 ms), long-duration (200 ms) red ganzfeld flashes on a rod-suppressing blue-adapting background [11]. After this first work, various types of stimulation and background conditions were tested.

Mortlock et al. [19] used white-on-white (W/W) stimulation. As expected, this resulted in a mass response of all cone photoreceptors [19, 20]. In contrast, monochromatic wavelength preferentially stimulated a single subtype of cone cells, inducing less spectral antagonism in the receptive fields of ganglion cells [20, 21]. Indeed, combinations of monochromatic stimuli (red 2.5 cd s/m^2^ on blue 10 cd/m^2^) probably minimize the centre-periphery antagonism of the receptive fields, thus resulting more effective in isolating the PhNR [20].

The spectral antagonism is due to the convergence of excitatory and inhibitory inputs from specific subtypes of cones. More specifically, the red-ON/green-OFF ganglion opposing cells receive both excitatory inputs from a long-wavelength-sensitive (L)-cone and inhibitory inputs from a medium-wavelength-sensitive (M)-cone, at the centre and periphery of the receptor field, respectively [22]. The signal that opposes L-cones to M-cones is transmitted through the population of midget ganglion cells, which are the most numerous [23], while the signal that opposes short-wavelength-sensitive (S)-cones to the LM-cones is transmitted through the bistratified ganglion cells, that are relatively few [22].

Since the S-cone cells present more permeable membranes [24] and higher metabolic demands, they are more sensitive to hypoxia and ischaemia [25, 26]. Thus, the S-cone (blue cone) PhNR of the ERG showed a high sensitivity in detecting glaucomatous damage [27, 28], similar to that provided by short wavelength automated perimetry (SWAP), which is a psycho-physical test that should detect visual field deficits 3–5 years before standard automated perimetry [29]. North et al. [30] hypothesized that the defect in the blue sensory mechanism could occur at a later stage of the glaucomatous disease, when the ganglion cell is no longer functioning. This was confirmed by the correlation found between SWAP and retinal nerve fiber layer (RNFL) thickness measured by optic coherence tomography (OCT) in glaucoma patients [30].

On the other hand, it is known that the PhNR tends to decrease with the shortening of stimulus wavelength, this being probably related to differential inductions of spectral antagonisms in the receptive fields of distinct RGC populations [21]. By eliciting the L-cones in an almost selective way [31], the red monochromatic stimulus predictably provides the greatest selective input to the centre or periphery of the receptive fields of the midget ganglion cells, as well as to the periphery of the bistratified ganglion cells, hence minimizing the spectral antagonism and resulting in better PhNR measurements. Indeed, the red flash stimulates the OFF-retinal pathways less efficiently, thus resulting in a lower b-wave when compared with a white stimulus, according to Rufiange et al. [32]. However, Rangaswamy et al. thought this could depend on the unequal summation of signals from the three subtypes of cones, with the b-wave amplitude resulting less affected by the red light stimulation than the white one [20].

In order to optimize clinical applications, the International Society for Clinical Electrophysiology of Vision (ISCEV) has recently provided a protocol for recording and analysing the PhNR in response to a brief flash. The ISCEV extended protocol advises, after pupil dilation and 10 min of photopic adaptation, to use short stimuli (< 5 ms) of red light (630–660 nm, 1.0–2.5 phot cd s/m^2^) on a background saturated with blue (450–485 nm, 10 phot cd/m^2^) [33] (Fig. 2).

## Measuring the PhNR: different techniques

Initially, there was a great variety of orientations in methodology to achieve the most accurate and reliable results in PhNR measurement.

In a study comparing open-angle glaucoma patients with healthy controls, Sustar et al. [21] decided to measure the PhNR from baseline to trough (BT) (Fig. 2). The Authors maintained the same blue background (470 nm, 10 cd/m^2^) and used different stimulus wavelengths (i.e. red, amber, green and white), HK-loop electrodes (in contact with the sclera at the conjunctival fornix), and 10 min of photopic adaptation. The best performance was obtained using a 2.5 cd s/m^2^ red stimulus (635 nm, 4 ms), while increasing the stimulus power (to 5 cd s/m^2^ and 7.5 cd s/m^2^, respectively) resulted in less reliable measurements due to glare artefacts. Moreover, a statistically significant difference in PhNR amplitude between healthy subjects and glaucoma patients was observed, regardless of using either a red or a white light stimulation. However, employing a red stimulus resulted more sensitive in terms of amplitude reduction (− 68%, *p* < 0.001) when compared with a white stimulus (− 38%, *p* = 0.001). Confirming this finding, the cutoff obtained with the red stimulus (i.e. − 16.2 μV) provided a 92.9% specificity and a 92% sensitivity, thus performing well in distinguishing between glaucomatous and healthy subjects.

In an effort to determine the most reliable technique for assessing the PhNR amplitude, Mortlock et al. [19] recorded ERGs in 31 healthy subjects (aged from 21 to 40 years) using both DTL electrodes and skin electrodes. The Authors used a 1.5 cd s/m^2^ red stimulus on a continuous blue background of sufficient intensity to saturate S-cones and rods (3.9 log scot td), after pre-adapting the subjects to the background luminance for a minimum of 5 min, and assuming a 7-mm pupil dilation. The following amplitude measurement techniques were used:

- PT (Peak-to-Trough, i.e. the difference between the peak of b-wave and the PhNR trough) (Fig. 2);
- BT (Baseline-to-Trough, i.e. the difference between the pre-stimulus baseline and the PhNR depression) (Fig. 2);
- BF (Baseline-to-Fixed time-point, i.e. the amplitude measured from the baseline to a predetermined time-point);
- PF (Peak-to-Fixed time-point, i.e. the difference between the peak of b-wave and a set time-point);
- PTR (ratio b-wave/PhNR, i.e. the ratio between b-wave amplitude—measured from the depression of a-wave to the peak of b-wave—and PhNR amplitude measured as PT).

As a result, the amplitude of the PhNR measured as PT seemed to achieve a better inter-ocular and inter-session repeatability (around 50% and 45% variability, respectively). Interestingly, they found PTR to provide the most repeatable results (around 25% variability for skin and DTL electrodes), this being particularly useful when the b-wave shows a reduced amplitude response. On the other hand, PhNR measurement as BT was the poorest performing technique and should be avoided according to the Authors. In case a prominent i-wave was present after b-wave (in most cases at larger amplitudes), the PhNR trough was identified as the lowest point, which may precede or follow the i-wave*.* Subsequently, the implicit time (IT) was measured at this trough (mean value of 71.5 ms). Therefore, in clinical terms, this study suggested that a longitudinal change in PTR value of more than 25% or an alteration of more than 45% in PT should be considered clinically significant, as well as any inter-ocular difference in PTR of more than 25%.

Viswanathan et al. found an increase in IT of about 8 ms when comparing a group of healthy elderly subjects (aged 71–80 years) with a group of younger controls (aged 21–30 years) [18]. In a subsequent work, Binns et al. [34] used the Naka-Rushton equation to evaluate the intensity–response ratio, measuring the PhNR both as PT and BT. This study identified PT as the best parameter for PhNR assessment in terms of variability [34].

It is worth noting that the neuronal alterations due to glaucoma affect the amplitude of the PhNR, leaving the implicit time relatively unchanged [18, 27, 35]. All components of the ERG tend to vary more in breadth than IT, and this also applies to the PhNR [19, 36]. However, it is important to keep in mind that inter-individual variations can be attributed to anatomical factors such as pigmentation of the fundus [37] and axial length [38, 39], or technical factors (e.g. electrodes position, impedance) [39], as well as intrinsic changes in retinal function.

Tang et al. [15] evaluated the reliability of the PhNR with the test–retest method in 49 healthy subjects (aged 21–72 years), choosing to investigate the right eye to decrease variability. After pupil dilation, signals were acquired using DTL electrodes, a pre-adaptation to background room light of at least 1 min, and a brief pre-adaptation to blue background (10 cd/m^2^) of approximately 1 min before delivering the first stimulus. This study followed the criteria previously adopted by Mortlock et al. [19], measuring BT, BF and PT, but also considering the BT/b-wave ratio. As a result, the most reliable measurements were obtained with a red stimulus (4 ms, 1 cd s/m^2^) and, in agreement with Mortlock et al.’s work [19], PT was demonstrated to be a reliable method in terms of repeatability. Furthermore, they found no statistical correlation between PhNR amplitude and age.

More recently, Van Alstine and Viswanathan explored the test–retest reliability of the multifocal PhNR (mfPhNR) using DTL electrodes [40]. They studied the right eye of 61 healthy subjects (aged 22–79 years) on two separate days. Based on previous mean IT measurements [19], the mfPhNR was recorded at a fixed time-point (75 ms) at the center of the macula considering a temporal interval of 15 ms. In agreement with Tang et al. [15], the Authors concluded that PT was the best performing measurement technique in terms of reproducibility when compared to BT, BT/PT ratio and BT/b-wave ratio. From a clinical point of view, since BT reflects RGCs activity and constitutes 30–50% of PT value, a reduction in PT of about 30–50% was considered a suspicious sign of disease.

Joshi et al. [41] also used the Naka–Rushton equation, which allows a quantitative description of the intensity–response function, in order to evaluate the effect of age and test–retest reliability of the PhNR in 45 healthy subjects. ERG was recorded using DTL electrodes, red stimuli (duration < 5 ms, intensity range 0.00625–6.4 cd s/m^2^) on a constant blue background (7 cd/m^2^). However, only data up to 1.6 cd s/m^2^ were used for the Naka–Rushton fit as a photopic hill phenomenon was observed not only in BT, PT and b-waves, but also in the PhNR [28, 34]. Therefore, they reported that none of the Naka–Rushton fit parameters were significantly affected by age for PT, BT or b-wave measurements. However, it has to be mentioned that BT measurements alone resulted in a weak but statistically significant correlation with age. With respect to test–retest reliability, the fit parameters for PT measurements performed better than those for BT. Previous studies [15, 19, 39] found that the test–retest repeatability of the BT/PT ratio in healthy subjects was better than that obtained with BT alone. On the other hand, BT reflects RGCs response and should be more indicative for glaucoma than PT, which also includes the activity of unaffected or slightly compromised bipolar cells. Thus, since the b-wave is generated by bipolar cell activity and glaucoma selectively affects RGCs, the normalized BT/b-wave ratio should theoretically better detect glaucomatous alterations. Supporting this, Preiser et al. showed that the BT/b-wave ratio was more sensitive to glaucomatous damage than BT alone [42]*.*

## Measuring the PhNR: clinical experiences

PhNR amplitude has been found to be reduced in many pathological conditions including glaucomatous optic neuropathy [43], ocular hypertension [18, 30, 44, 45], Leber hereditary optic neuropathy [46], optic nerve atrophy [47], diabetic retinopathy [48], central retinal artery occlusion [49] and idiopathic endocranial hypertension [50, 51]. After having established that the red-on-blue (R/B) stimulation gives more discriminating results than white-on-white (W/W) stimulation in detecting glaucomatous damage [20, 21], we now present the most interesting experiences in the field.

In a prospective, cross-sectional study Banerjee et al. [52] compared 25 glaucoma patients with 50 age-matched healthy controls, using Burian-Allen electrodes, 10 min of pre-adaptation to blue, white and yellow backgrounds (10 cd/m^2^), and various stimulus intensities in a first phase. Then, they used the following settings as reference parameters: stimulus duration < 4 ms, 3.5 cd s/m^2^ intensity for R/B and W/W stimulations, 1 cd s/m^2^ intensity for B/Y. When employing the R/B method, PhNR amplitude was found to be significantly reduced in glaucoma eyes (27.11 ± 14.88 µV), while IT was increased (77.98 ± 6.37 ms). Receiver operating characteristic (ROC) curve was performed for all stimuli in order to analyse the area under the curve (AUC) and sensitivity–specificity. The ROC curve showed largest AUC in R/B PhNR and also higher sensitivity and specificity (72% and 80%, respectively). Interestingly, the specificity of red-on-blue PhNR was found to be better than that of RNFL thickness and slightly better than that of MD. Even though the amplitudes recorded in the B/Y ERG of glaucomatous patients were much higher in absolute value than in controls (even using very low intensities in this setting), the corresponding B/Y PhNR reduction was less pronounced than that obtained with R/B stimulation.

Cvenkel et al. [53] evaluated the discrimination ability of both PERG and PhNR in patients with ocular hypertension (OHT) and early glaucoma as well as in glaucoma suspects, comparing them with healthy controls. According to the Authors, PhNR amplitude measured from the baseline (BT) distinguished best between glaucoma and control groups and performed better than PhNR/b-wave ratio. Of note, in eyes with suspected glaucoma, decrease in PhNR amplitude was associated with small changes in peripapillary retinal and macular NFL thicknesses. However, PhNR showed a high sensitivity (91.7%) for both glaucoma suspect and early glaucoma, but lower specificity (58.8% and 70% in the two groups, respectively). In other words, approximately one-third of healthy subjects could be recorded as false positive.

Preiser et al. [42] examined PhNR and steady-state PERG from patients with pre-perimetric glaucoma (defined as the presence of pathological optic disc appearance and normal visual field) and glaucoma, comparing them with a control group. PhNR was recorded using a red stimulus (5 ms, 635 nm) on a blue background (450 nm, 10 cd/m^2^), and varying the intensity from 0.1 to 4 cd s/m^2^. Then, the PhNR amplitude from baseline to trough at 72 ms and the PhNR/b-wave ratio were measured. Both PhNR and PERG performed similarly to distinguish between healthy subjects and pre-perimetric patients. In particular, both PhNR/b-wave ratio and 0.8°/16° ratio of steady-state PERG performed better than amplitudes. However, no correlation was found between the two ratios, while there was a correlation between the PhNR and the 0.8° PERG. Since the PERG can be affected in eyes with pre-perimetric glaucoma [54–57], and the PhNR seems to be more closely related to perimetric defects [12], the corresponding ratio values might detect different disease mechanisms.

Niyadurupola et al. [58] evaluated glaucomatous patients in which the intraocular pressure (IOP) dropped by more than 25% compared to the baseline. This study found a correlation between IOP decrease, and both PhNR amplitude increase and PhNR/b-wave ratio. Signals were recorded using DTL electrodes and stimuli with intensities similar to those subsequently recommended by ISCEV [33] (2.25 and 3 cd s/m^2^, blue background of 20 cd/m^2^). Using a brief red stimulus (2.25 cd s/m^2^), the recorded PhNR was higher in healthy controls (27 ± 3.5 μV) when compared to OHT and glaucomatous patients (18.5 ± 2.8 μV and 12.7 ± 1.6 μV, respectively). Similarly, the PhNR/b-wave ratio also resulted higher in healthy subjects (0.319 ± 0.035) than OHT and glaucoma patients (0.269 ± 0.051 and 0.134 ± 0.016, respectively). Using HK-loop electrodes and R/B method (2.5 cd s/m^2^, 10 cd/m^2^), similar results to those found by Sustar et al. [21] were reported: the measured PhNR was 25.4 ± 7.1 μV and 8.3 ± 5.8 μV in healthy controls and glaucomatous patients, respectively.

With similar parameters (red stimulus of 2 cd s/m^2^ on blue background of 25 cd/m^2^), Shen et al. [45] reported different PhNR values, namely 47.8 ± 10.7 μV and 27.2 ± 13.5 μV in healthy controls and glaucoma patients, respectively. These results were close to those reported by Banerjee et al., who used a stronger stimulus (3.5 cd s/m^2^) [52]. However, the Authors specified neither which type of corneal electrodes was used nor reported any IT measurement.

Machida et al. [59] used a red stimulus (3 ms, 644 nm, 1600 cd/m^2^) on a blue background (470 nm, 40 cd/m^2^) in open-angle glaucoma patients and healthy subjects. By analysing the PhNR amplitude (measured from baseline to trough at ~ 70 ms) and the PhNR/b-wave ratio, they found a significant correlation with RNFL thickness measured by OCT and optic disc topography (rim area, cup/disc area ratio) assessed by confocal scanning laser ophthalmoscopy. More specifically, the PhNR amplitude decreased together with the functional worsening of glaucoma (in terms of visual field defect), as well as the neural loss assessed by RNFL and optic nerve head morphology. Furthermore, differences between optic nerve atrophy (induced by trauma, compression and optic neuritis) and glaucomatous disease were investigated. In a previous work by Gotoh et al. [47], PhNR amplitude highly correlated with RNFL thickness, which showed diffuse and thus more easily detectable damage. On the other hand, this correlation was much lower in glaucomatous eyes because the localised RNFL impaired areas were more difficult to detect at early stage of disease. In agreement with Shen et al. [45] and considering that the PhNR is supposed to reflect the overall function of the RGCs throughout the entire retina, PhNR and RNFL measurement seemed more suitable for optic nerve pathology assessment. Indeed, in these cases RGCs are more widely impaired than in early or moderate stages of glaucoma, where RGCs are only regionally affected. Hence, in subsequent works, more attention was focused to focal techniques alone [12, 44, 60–63].

It is also worth mentioning Wilsey et al.’s study on non-human primate models of experimental glaucoma [64]. The Authors compared diagnostic performance and structure–function correlations of multifocal ERG (mfERG), full-field flash ERG (ffERG) PhNR and transient PERG. Based on previous evidence, the high-frequency component (HFC) of the slow-sequence of mfERG, the PhNR amplitude (measured as BT) of the red-on-blue ffERG and the N95 of the transient PERG were considered as the best parameters from each mode in terms of diagnostic efficacy and correlation to anatomic damage. It is important to note that these parameters represent three different expressions of RGCs activity, as the N2 from mfERG is comparable to the PhNR from ffERG, which shares in turn the same generators of the N95 from PERG [13, 65]. The findings confirmed the high sensitivity of PhNR and PhNR/b-wave ratio in detecting RGCs functional impairment, as well as the strong correlation with structural loss and the best overall diagnostic accuracy. The normalized HFC showed similar results, but the mfERG was shown to provide only modest diagnostic support in human glaucoma [57, 66–70]. On the other hand, the transient PERG showed the widest population variance and the poorest test–retest reliability, thus performing worse than the other two ERG modalities. However, it should be kept in mind that different ERG parameters might detect complementary diagnostic information. The Authors addressed this question by plotting the eyes flagged by each of the best performing ERG parameters under study in area-proportional Euler diagrams. Since some cases were detected only by one parameter (8/42 by mfERG,5/42 by PhNR,1/42 by PERG), these results suggested that complementary information could be available through testing by multiple ERG modalities.

## Measuring the PhNR: pros and cons in clinical practice

As an objective measure of global retinal function, ERG testing is a diagnostic tool with several potential applications in ophthalmology, even in pathologies that clinicians manage on a daily basis. However, its use is sometimes associated with niche disease entities and there is often a need to refer patients to a specialised centre for testing and interpretation. As a result, there may be a perception that the technique is impractical for routine clinical practice. From this point of view, the availability of office-based platforms including tabletop, cart-based and suitcase-sized units might allow routine access to different types of electrophysiology testing and change the perceived clinical utility of ERG and PhNR. Moreover, the lack of a standardized technique for PhNR clinical testing has been recently addressed with the publication of the ISCEV-extended protocol [33].

Currently, the PERG is the most well-established ERG technique for glaucoma detection [71–75], and previous studies indicated that it may be most beneficial as an adjunct in the diagnosis and management of glaucoma suspects (with normal or borderline visual fields and/or RNFL thickness) by helping stratify the risk [76, 77]. However, careful control of steady central fixation, optimal refractive correction and clear ocular media are necessary. More importantly, as it depends on a cascade of intact outer retinal signals, the PERG alone is not a specific assay of RGC function and needs to be “confirmed” by a ffERG to specifically evaluate the macular cone and cone bipolar responses. On the other hand, the PhNR may be able to overcome these limitations, being potentially more feasible in a clinical setting. Even relying on a preserved feed-forward response of cone photoreceptor and cone bipolar cells as PERG, the PhNR gives an immediate way of assessing the outer retinal cells functional state, by simultaneously recording a- and b-waves and without any other confirmation test. Furthermore, the PhNR should allow an early detection of functional abnormalities of all the RGCs including the most peripheral ones, whereas the PERG examines only the central RGCs, which are involved later in the development of manifest (or perimetric) glaucoma. This means that the PhNR amplitude may reveal alterations in eyes with a normal visual field, such as OHT or suspect glaucoma, while the N95 wave of PERG tends to decrease as the disease progresses affecting the ganglion cells within the central 30° of the visual field. Furthermore, in addition to its independence of patient motor and cognitive skills (unlike standard automated perimetry), the PhNR does not require refractive correction, meticulous fixation monitoring or ocular media transparency (which is also important for reliable fundus photography and OCT acquisition).

A possible clinical disadvantage might be represented by the need for pupil dilation to improve PhNR measurement accuracy. However, since a comprehensive dilated eye examination should be performed routinely in almost any patient—and even more crucially in glaucoma suspects to correctly assess the optic nerve, the PhNR could be recorded at the end of the visit. This could allow clinicians to have an additional element supporting a diagnosis and guiding appropriate treatment decisions in uncertain cases such as glaucoma suspects or ocular hypertensive subjects.

## Conclusion

The main points of this review are summarized in Table 1. The most reliable PhNR measurements are obtained using a red stimulus on a blue background. Considering its direct correlation with retinal ganglion cells response, the PhNR measured as baseline-to-trough (BT) represents the most reliable parameter in representing RGCs activity. On the other hand, using a predetermined time-point for recording signals does not seem appropriate because of age-related implicit time (IT) variability.

**Table 1:** Main points

The photopic negative response (PhNR) of the cone-driven ERG is a slow negative wave that follows the b-wave and originates from the innermost layers of the retina
The PhNR is an objective measure of retinal ganglion cells (RGCs) functional status and sensitive to glaucomatous alterations
A monochromatic red stimulus on blue background (R/B) results more effective in isolating the PhNR
The PhNR test does not require refractive correction, meticulous fixation monitoring or ocular media transparency
A reduced PhNR may play a potential role in early glaucoma detection, risk stratification and disease progression monitoring, especially when visual field testing and/or OCT measurement are inconclusive, not reliable, or difficult to perform

It is well known that the current “gold standard” for glaucoma diagnosis and monitoring is a combination of stereoscopic assessment of the optic disc, evaluation of structural changes in the optic nerve detected by OCT and visual field tests. Nevertheless, it has been shown that glaucoma patients with manifest visual field defects show pathologic PhNR values, and the PhNR seems promising in detecting early glaucoma. However, distinguishing between healthy subjects and suspect patients at risk of developing glaucomatous damage still remains challenging. Thus, comparing reports of the routine examinations (optic disc assessment, OCT RNFL measurement, standard automated perimetry) with the results of electrophysiological examinations might be helpful in the differential diagnosis of uncertain clinical cases. In particular, the PhNR and the steady-state pattern ERG optimized for glaucoma screening (PERGLA) can explore the parvocellular and magnocellular pathways, respectively. The combined peculiarities of these two techniques could give ophthalmologists a useful feedback to detect early alterations suggestive of glaucomatous pathology and stratify the risk. Furthermore, the PhNR could also represent a useful additional tool in monitoring the progression of glaucomatous disease. One of the main limitations in previous studies investigating the role of PhNR in glaucoma was the lack of a standardized technique in assessing this ERG component. However, the ISCEV has now provided a way to partially overcome this issue by publishing its extended protocol for recording and analysing the PhNR.

In conclusion, we believe that the PhNR may be worthy of more clinical consideration, and further research following international guidelines and involving larger populations in longitudinal studies might gain more insight into glaucoma pathophysiology as well as early detection of disease and risk assessment.

## Data Availability

Not applicable.
